# CERA Detection and Stability in Blood Versus Urine

**DOI:** 10.1002/dta.3960

**Published:** 2025-10-21

**Authors:** Olivier Salamin, Joséphine Chappuis, Lasse V. Bækken, Tiia Kuuranne, Nicolas Leuenberger

**Affiliations:** ^1^ Swiss Laboratory for Doping Analyses, University Center of Legal Medicine, Lausanne and Geneva Lausanne University Hospital and University of Lausanne Lausanne Switzerland; ^2^ Nordic Athlete Passport Management Unit, Norwegian Doping Control Laboratory, Department of Pharmacology Oslo University Hospital Oslo Norway

## Abstract

Erythropoietin receptor agonists (ERAs), including continuous erythropoietin receptor activators (CERAs), are potent blood doping substances used to enhance endurance performance by stimulating erythropoiesis. While traditionally detected through direct analysis of urine or serum samples using sarcosyl‐polyacrylamide gel electrophoresis (SAR‐PAGE) and western blotting, the slow urinary elimination of third‐generation ERAs like CERA has shifted anti‐doping strategies toward serum‐based detection. This study compared the detectability and stability of CERA in urine and serum matrices and evaluated the added value of combining direct detection with hematological profiling. Using samples from a controlled CERA administration study and an authentic case example, we assessed CERA detection in serum, urine, and simulated dried blood spot (DBS) matrices (Tasso‐M20). Additionally, we conducted stability experiments by incubating spiked matrices at 37°C for up to 72 h. Our results confirmed the superior stability and consistent detectability of CERA in serum and DBS compared with urine. Moreover, hematological alterations such as increased reticulocytes percentage flagged by the Athlete Biological Passport (ABP) supported targeted serum testing, leading to the successful detection of CERA. These findings highlight the importance of systematic blood collection for both direct and indirect detection strategies. Furthermore, DBS samples showed promising analytical performance and resistance to elevated temperature, suggesting their utility as minimally invasive alternatives in anti‐doping programs. Overall, our study reinforces the relevance of blood matrices in the detection of CERA and advocates for the broader integration of blood‐based strategies in targeting doping practices with ERAs.

## Graphical abstract

This study presents comparative data on CERA detection in serum and urine samples, highlighting adverse analytical findings (AAFs) in serum that were undetected in corresponding urine samples. This shift would significantly improve the efficiency and sensitivity of CERA detection in doping control, ensuring more effective doping control programs.

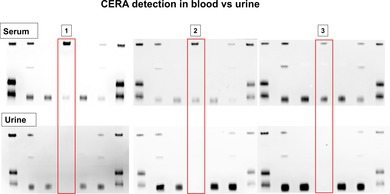

## 1 Introduction

Blood doping aims to increase hemoglobin mass and the number of circulating red blood cells, thereby enhancing oxygen delivery to working muscles and improving endurance performance. This is typically achieved through two main strategies: blood transfusion (either homologous or autologous) or administration of erythropoietin receptor agonists (ERAs). These compounds mimic the action of endogenous erythropoietin (hEPO) by binding to and activating EPO receptors in the same manner as the native hormone, thereby stimulating the proliferation of immature erythrocytes in the bone marrow. They are produced by recombinant DNA techniques using various mammalian cell lines and differ only slightly from endogenous EPO, mainly due to variations in glycosylation and sulfation patterns.

Over time, three main generations of ERAs have been developed with the therapeutic aim of improving the treatment of anemia by enhancing efficacy and prolonging half‐life to improve patient compliance [1]. First‐generation ERAs (known as recombinant EPO—rEPOs), such as epoetin alfa and epoetin beta, are structurally very similar to endogenous EPO and require frequent administration due to their short half‐life. Second‐generation products, such as darbepoetin alfa, contain additional glycosylation sites, resulting in an extended half‐life and less frequent dosing. Third‐generation agents, including continuous erythropoietin receptor activators (CERAs) like methoxy polyethylene glycol‐epoetin beta, feature large modifications such as PEGylation, which significantly prolongs circulation time in blood and alters receptor interaction dynamics, further extending their pharmacokinetic profiles.

The addition of the PEG moiety also markedly increases the molecular weight, reducing glomerular filtration and limiting urinary excretion [2]. As a result, blood has become the preferred matrix for CERA detection in the anti‐doping context. While CERA may not be the most commonly misused ERA due to its slow elimination and prolonged detection window, it could be selected for doping practices due to its superior detectability in blood rather than urine, which remains the most frequently collected matrix during antidoping tests. Several antidoping investigations have confirmed that CERA continues to be relevant in doping scenarios, particularly in endurance sports. Notably, reported cases often refer to targeted efforts where blood samples were collected alongside urine, highlighting the critical role of serum testing [3].

Direct detection of ERAs, including CERA, relies on a technique using sarcosyl‐polyacrylamide gel electrophoresis (SAR‐PAGE) and western blotting, which is capable of distinguishing recombinant from endogenous EPO based on molecular weight, either in urine or blood [2]. However, due to the complexity and time required for these analyses, they are not routinely applied to all doping control samples. Instead, they are typically requested for cases with specific suspicion, often prompted by findings from the indirect detection approach such as the hematological module of the Athlete Biological Passport (ABP). The ABP monitors longitudinal changes in markers such as hemoglobin and reticulocyte percentage to detect abnormalities suggestive of blood doping [4]. In some instances, ABP data alone can establish an anti‐doping rule violation, while in others, specific fluctuations in any of the biomarkers may guide targeted follow‐up with direct detection methods, increasing the success of identifying doping practices. These specific requests are often recommended by the Athlete Passport Management Units (APMU), which are responsible for the evaluation of each new sample added to an athlete's profile [5].

Because both the ABP and direct detection method for ERAs benefit from blood‐based analysis, the systematic collection of blood samples plays a crucial role in anti‐doping programs [6]. In addition to enabling longitudinal monitoring, the blood matrix offers a practical advantage for direct EPO detection: endogenous EPO is stable and consistently detectable in serum, providing a reliable baseline for comparison [7]. In contrast, urine samples are prone to alterations due to external (e.g., transit time and temperature) or internal factors (microbial activity, in‐competition event) and can occasionally show undetectable or weak endogenous EPO signals, complicating the interpretation of analytical results. Recent work by Miller et al. further supports the robustness of blood‐based EPO testing, demonstrating that both endogenous and rEPO remain stable in microvolumetric capillary serum samples even when shipped at ambient temperature [8]. This further highlights the value of including more blood collections in doping control strategies, particularly when targeting EPO‐based doping. In this context, alternative matrices such as dried blood spots (DBSs) may also offer practical advantages regarding EPO stability and enabling minimally invasive collection, easier storage, and potential for retrospective analysis, further expanding the toolkit for ERAs detection and monitoring.

In this study, we compared the detection and stability of CERA in urine and serum matrices. Additionally, we explored the relationship between CERA detection and the hematological changes it triggers, using a case example to illustrate the added value of combining direct and indirect detection strategies.

## 2 Material and Methods

### 2.1 Detection of Endogenous and Recombinant Human EPOs Using SAR‐PAGE and Western Blotting

Samples were analyzed following the WADA guidelines in effect at the time of testing to differentiate endogenous EPO (hEPO) and ERAs [9]. Serum and Tasso‐M20 samples were processed using EPO Purification Gel Kits (EPGK) for blood, incorporating the monoclonal antibodies 3F6 and 7D3 (MAIIA Diagnostics, Sweden), following the manufacturer's instructions. For serum samples, 500 μL of serum was mixed with 5 mL of sample buffer and loaded onto the purification column via an attached funnel. Samples were incubated end‐over‐end at room temperature for 120 min. The flow‐through was filtered through the column, which was then washed, and hEPO along with ERAs was eluted using 50 μL of elution buffer by centrifugation at 300*g* for 1 min. The eluate was subsequently concentrated to approximately 5 μL using an Amicon 30‐kDa molecular weight cut‐off filter (Merck Millipore Ltd, MA, USA) at 14,000*g* for 10 min. Immunopurified samples were then combined with 15 μL of loading buffer, and the samples were stored at −20°C until analysis. Tasso‐M20 samples were extracted using the same procedure with the 7D3 monoclonal antibodies, applying a single pod corresponding to approximately 17.5 μL of blood.

Urine samples were purified using the EPGK for Urine kit and the 3F6 antibody (MAIIA Diagnostics, Sweden). A sample buffer was added to 10 mL of urine and incubated for 10 min. The samples were then incubated end‐over‐end at room temperature for 120 min. After washing the column, hEPO and ERAs were eluted with 50 μL of elution buffer by centrifugation at 300*g* for 1 min. Immunopurified samples were then combined with 15 μL of loading buffer, and the samples were stored at −20°C until analysis.

Following purification, hEPO and ERA proteins were separated using NuPAGE 10% Bis‐Tris Midi polyacrylamide gels (Thermo Fisher Scientific) as described in [10]. Separation was performed at 125 V for 3 h using SAR running buffer. The samples were then transferred to an Immobilon‐P PVDF membrane for 45 min; then the membrane was blocked by agitation in 5% skim milk for 1 h. Between these steps, the membrane was washed in PBS with three 30‐s washes and one 10‐min wash. The membrane was then incubated overnight in biotinylated anti‐EPO antibody (AE7A5, 0.5 μL/mL 1% milk); incubated 1 h at RT in streptavidin‐HRP complex; and washed again. Finally, images of the membrane were acquired using a maximum sensitivity chemiluminescent substrate (SuperSignal West Femto, Thermo Scientific) and an Amersham Imager 800 camera. Results were then processed using GASepo software, Version V2.1.2.15476.

### 2.2 Preparation of Tasso‐M20 Samples From CERA Administration Study

Serum samples from a single subject were obtained from a pilot excretion study by Lamon et al., in which healthy male volunteers received a single 200 μg injection of CERA, with samples collected at regular intervals up to 27 days post‐injection [11]. These samples were used to prepare modeled‐blood Tasso‐M20 spots as previously described [12, 13].

In brief, erythrocytes were isolated from a non‐treated subject by centrifuging freshly collected blood in EDTA tubes at 1500*g* for 15 min, followed by two washes with 0.9% NaCl (hypotonic saline). The washed erythrocytes were then gently mixed in a 1:1 ratio with each serum sample from the administration study to simulate whole blood. A 17.5 μL aliquot of this reconstituted blood was applied to a Tasso‐M20 volumetric pod and allowed to dry at room temperature for at least 4 h. The DBS were subsequently stored at 4°C with desiccant until analysis. Samples were extracted and analyzed as described above.

### 2.3 Stability Tests

To assess the stability and detectability of CERA, different biological and control matrices were spiked with CERA at a concentration of 0.1 μg/mL. The matrices included urine from three male and three female subjects, plasma from one subject, human serum (H4522‐20ML, Sigma‐Aldrich), pig serum (Porcine Serum, 26250‐084, Gibco by Life Technologies), phosphate‐buffered saline (PBS), and synthetic urine. All spiked samples were stored at 37°C for 0, 24, 48, and 72 h in an oven with controlled temperature. Rat erythropoietin (500 pg/sample) was added as an internal standard to each sample, following the protocol described by Zhou et al. [14], to normalize CERA signal intensity across gel lanes. Sample extraction and analytical procedures were carried out as described above. Additionally, modeled‐blood Tasso‐M20 spots prepared from the sample collected 27 days post‐CERA administration were also incubated at 37°C for 24, 48, and 72 h to assess CERA stability under the same conditions.

### 2.4 Hematological Profiling for Targeted Detection of CERA With the ABP

Indirect detection of EPO use and targeting for ERA analysis in the corresponding urine and serum samples was conducted through the hematological module of the ABP. Venous blood samples were collected in EDTA tubes under standardized pre‐analytical conditions and analyzed within the required timeframe. Hematological parameters including hemoglobin concentration (HGB) and reticulocyte percentage (RET%) were measured using an automated hematology analyzer (Sysmex XN‐1000, Sysmex Corp., Japan) in accordance with WADA requirements (WADA Technical Document—TD2021BAR, 2021). Additionally, the OFF‐score combining HGB and RET% was calculated (HGB (g/L) – 60 × √RET%). An individual longitudinal profile was generated and managed within the Anti‐Doping Administration and Management System (ADAMS) using a Bayesian adaptive model generating individual upper and lower limits. Atypical passport findings (ATPFs) or flags were automatically generated when a hematological parameter fell outside the calculated individual reference limits, potentially indicating blood manipulation, such as the use of ERA. The profile was reviewed by the APMU, which provided specific recommendations for further analysis and targeted testing following the addition of each new data point.

## 3 Results and Discussion

In 2024, 24% of the adverse analytical findings (AAFs) for ERAs reported by the Lausanne laboratory were attributed to CERA. These results highlight the risk associated with the misuse of CERA. Notably, 47% of all positive samples that year were derived from the serum matrix. Because urine remains the predominant matrix collected in anti‐doping programs, an imbalance in testing matrices may be strategically exploited by athletes to avoid detection. Similar trends have been documented in previous investigations: for example, a targeted operation in Guadeloupe revealed a high incidence of CERA‐positive cases in serum samples, highlighting matrix selection as a key factor in detection outcomes [3]. Furthermore, during the 2017 Vuelta a Costa Rica, 12 athletes tested positive for CERA, emphasizing the compound's continued relevance in doping practices [15]. Collectively, these observations highlight the critical need to include serum sample collection, particularly in targeted testing scenarios, to improve the detection of CERA and other ERAs.

**FIGURE 1:**
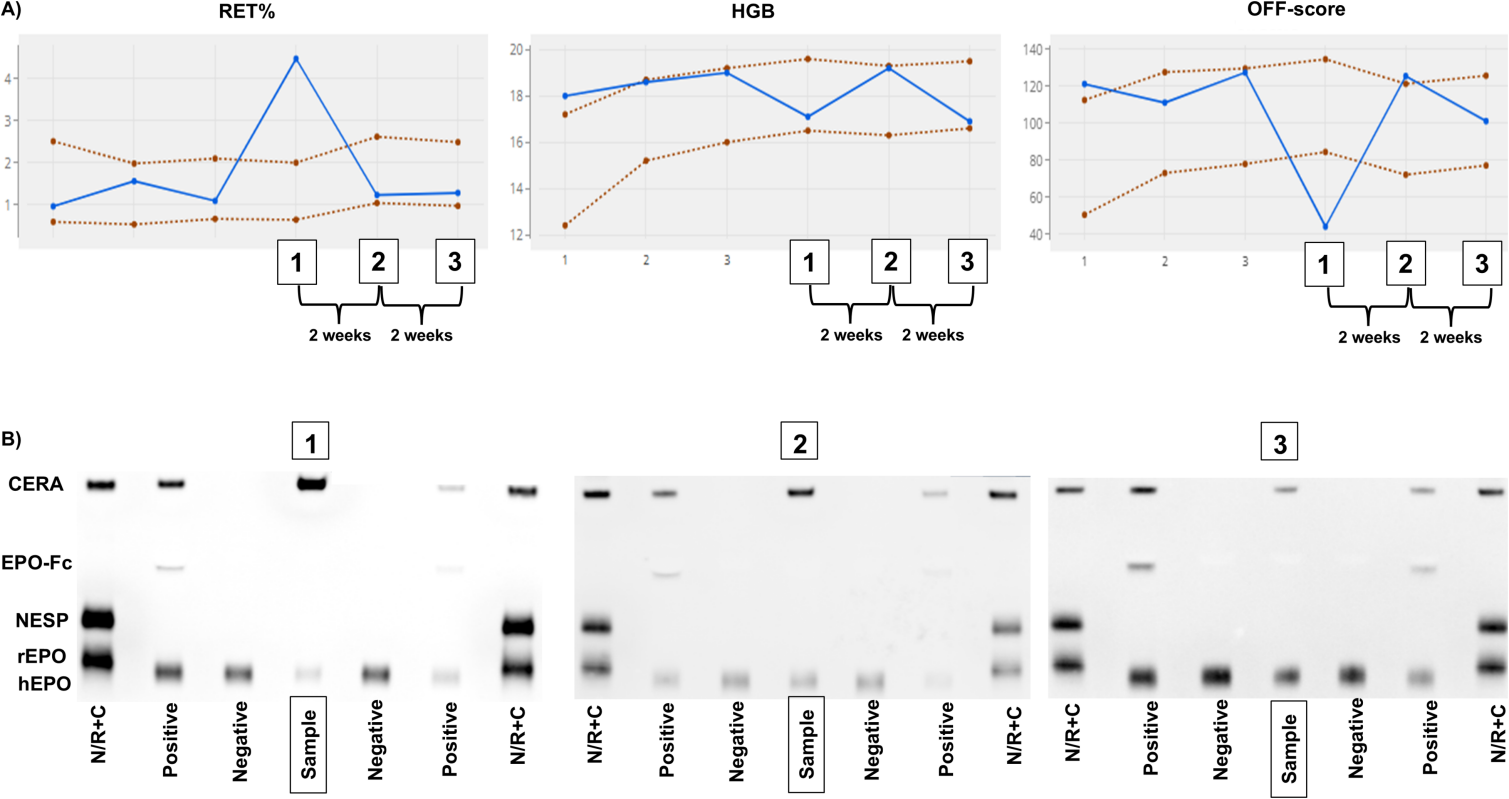
Comparative analysis of indirect (ABP) and direct detection approaches for three adverse analytical findings (AAFs) related to CERA. (A) Longitudinal hematological profile from the Athlete Biological Passport (ABP) of an athlete with three venous blood samples (1, 2, and 3), showing abnormal variations in key biomarkers, including hemoglobin concentration (HGB), reticulocyte percentage (RET%), and OFF‐score—suggestive of exogenous erythropoiesis‐stimulating agent (ESA) use. (B) Direct detection of CERA in the corresponding serum samples (1, 2, and 3) using SAR‐PAGE, showing a migration pattern consistent with CERA's high molecular weight.

Among the positive CERA findings, subsequent communication with the testing authority revealed that three originated from the same athlete. This athlete was tested three times at two‐week intervals with serum, urine, and whole blood collected at each sample collection session. To investigate the targeting scenario and the corresponding hematological responses of this particular athlete, the APMU was contacted to share the individual profile, with the consent of the passport custodian. It also allowed for combining the data and comparing between direct detection of CERA via SAR‐PAGE and indirect detection through the ABP (Figure 1).

For the first corresponding whole blood sample (#1) associated with the initial detection of CERA in serum, a significant RET% increase well above the upper individual limit was observed, paired with a decrease in the OFF‐score (Figure 1A). The immature reticulocyte fraction (IRF) also displayed an elevated value (data not shown). This observation indicated ongoing erythropoietic stimulation, prompting the APMU to request ERA analysis in the concurrently collected urine and serum samples, along with a recommendation for a prompt follow‐up test. The sample collected 2 weeks later (#2) showed a return of RET% to baseline levels and increased HGB, although not different from earlier samples, resulting in an OFF‐score exceeding the athlete's upper individual limit, although not significantly. An additional sample (#3) collected another 2 weeks later showed a return of all parameters to values within the individual limits.

Analysis of the serum from Sample #1 demonstrated the presence of CERA, characterized by a strong signal at the expected CERA migration height (Figure 1B) and a weak endogenous EPO signal, consistent with suppressed physiological production due to negative feedback. The two following serum specimens (Samples #2 and #3) also displayed clear CERA signals. Interestingly, in Sample #3, the relative signal intensities of CERA and endogenous EPO appeared inverted, suggesting a single dose of CERA before Sample #1 and a recovery of endogenous EPO production along with the decay of the exogenous stimulation.

Taken together, these results suggest that CERA can be detected in serum over an extended period using SAR‐PAGE as a direct detection method. Given that the peak erythropoietic response in RET% typically occurs 5–7 days after CERA injection [16, 17], the window for direct detection in this case can be extrapolated to exceed 30 days. In terms of indirect detection, had Sample #1 not been collected during the period of accelerated erythropoiesis, the overall profile may have been assessed as low priority, at least for the RET% profile, assuming the same doping regime (i.e., no continued CERA administration). Especially, no clear negative feedback is visible after the peak seen in Sample #1, as would be expected following repeated ERA administration [18] but not necessarily after a single CERA injection [11, 16]. Thus, the detection window was in this case longer for the direct method than for the indirect markers. This case highlights the strength of combining indirect markers and direct detection, as well as the importance of considering direct detection even when the hematological profile is not flagged by the adaptive model.

**FIGURE 2:**
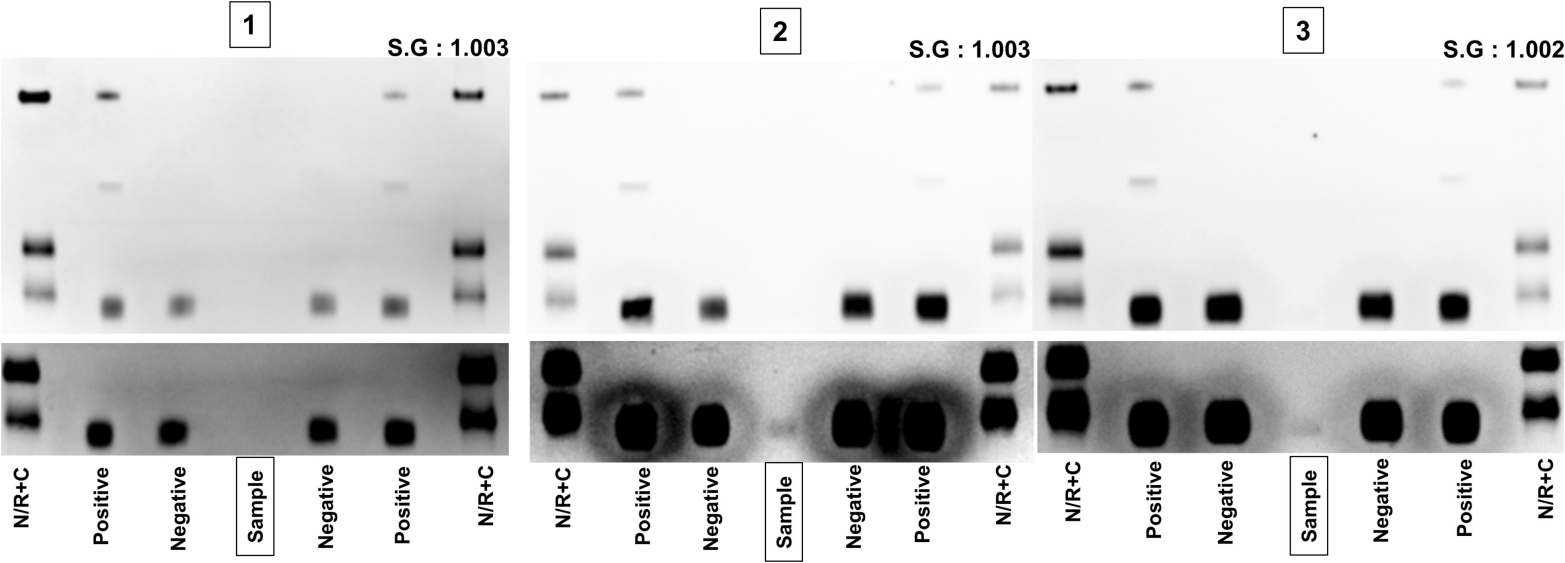
SAR‐PAGE analysis of the corresponding urine samples of serum positive for CERA. Urine samples 1, 2, and 3, corresponding to serum samples with confirmed CERA AAF, showed no detectable signal by SAR‐PAGE analysis. The lower panel displays the enhanced chemiluminescence detection. S.G.: specific gravity.

As recommended by the APMU and requested by the testing authority, the corresponding urine samples were also analyzed for ERA detection (Figure 2). All three urine samples resulted in negative findings for CERA. In Sample #1, in addition to the absence of the CERA signal, the electrophoretic band for hEPO was also absent. In Samples #2 and #3, a faint hEPO signal became visible after contrast enhancement using GASepo software. This aligned with the serum findings, where the hEPO signal showed partial recovery in Sample #2 and more substantial recovery in Sample #3. It is also worth noting that all three urine samples exhibited very low specific gravity, indicating diluted urine, which may have further complicated the detection of both hEPO and CERA. Finally, analysis of the steroidal profile revealed no clear indication of bacterial contamination in any of the three urine samples.

The results of this comparative analysis demonstrate differences between blood and urine matrices in the detection of CERA. The discrepancy is most likely attributed to the physicochemical properties of CERA, notably its high molecular weight resulting from PEGylation, which substantially reduces its glomerular filtration and consequently limits its renal excretion. While these case findings support that urine is not the optimal matrix, previous controlled administration studies have shown that CERA can still be detected in urine under specific conditions, such as in samples with high protein content or post‐exercise urine where protein excretion is increased [16, 19] The findings of this case highlight the importance of integrating serum testing in antidoping programs. In line with the prevailing EPO technical document, serum was proven more sensitive over urine matrix variability and capable of improving the overall success for the detection of CERA and ERA in general [9].

**FIGURE 3:**
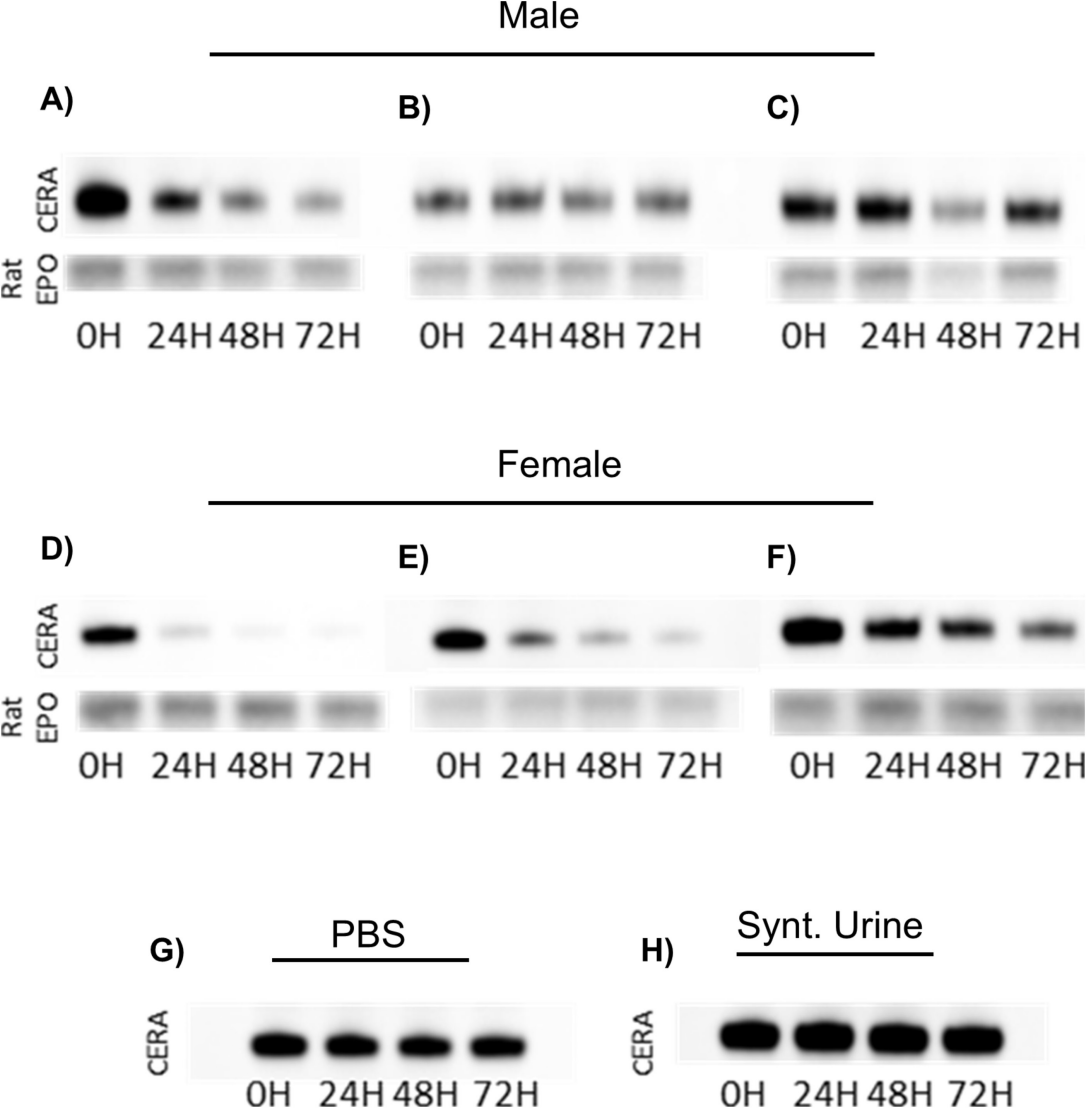
CERA instability in urine under thermal stress. CERA‐spiked urine samples from three male (A–C) and three female donors (D–F), phosphate‐buffered saline (PBS; G), and synthetic urine (H) were incubated at 37°C and analyzed after 0, 24, 48, and 72 h. Rat EPO served as an internal standard.

Beyond its limited glomerular filtration, the potential instability of CERA in the urinary matrix has been hypothesized as another factor that may affect its detectability. To explore this hypothesis, different biological matrices spiked with CERA were subjected to elevated temperature conditions (37°C) for up to 72 h. CERA generally showed signs of degradation in human urine when stored at 37°C (Figure 3), with the effect being especially pronounced in samples from female individuals, where only weak CERA signals remained after 72 h (Figure 3D,E). In contrast, CERA remained remarkably stable in PBS and synthetic urine under identical conditions (Figure 3G,H). This observation suggests that CERA degradation is mediated by urine‐specific factors. Moreover, the more pronounced instability observed in female urine samples could be related to a higher bacterial load, a phenomenon previously reported in the literature [20]. However, a limitation of this study is the small number of urine samples analyzed (three female and three male), which do not capture the full variability of urinary composition across individuals; further investigations on larger cohorts, particularly with additional male samples, will be required to assess whether CERA degradation may also occur under different urinary conditions.

**FIGURE 4:**
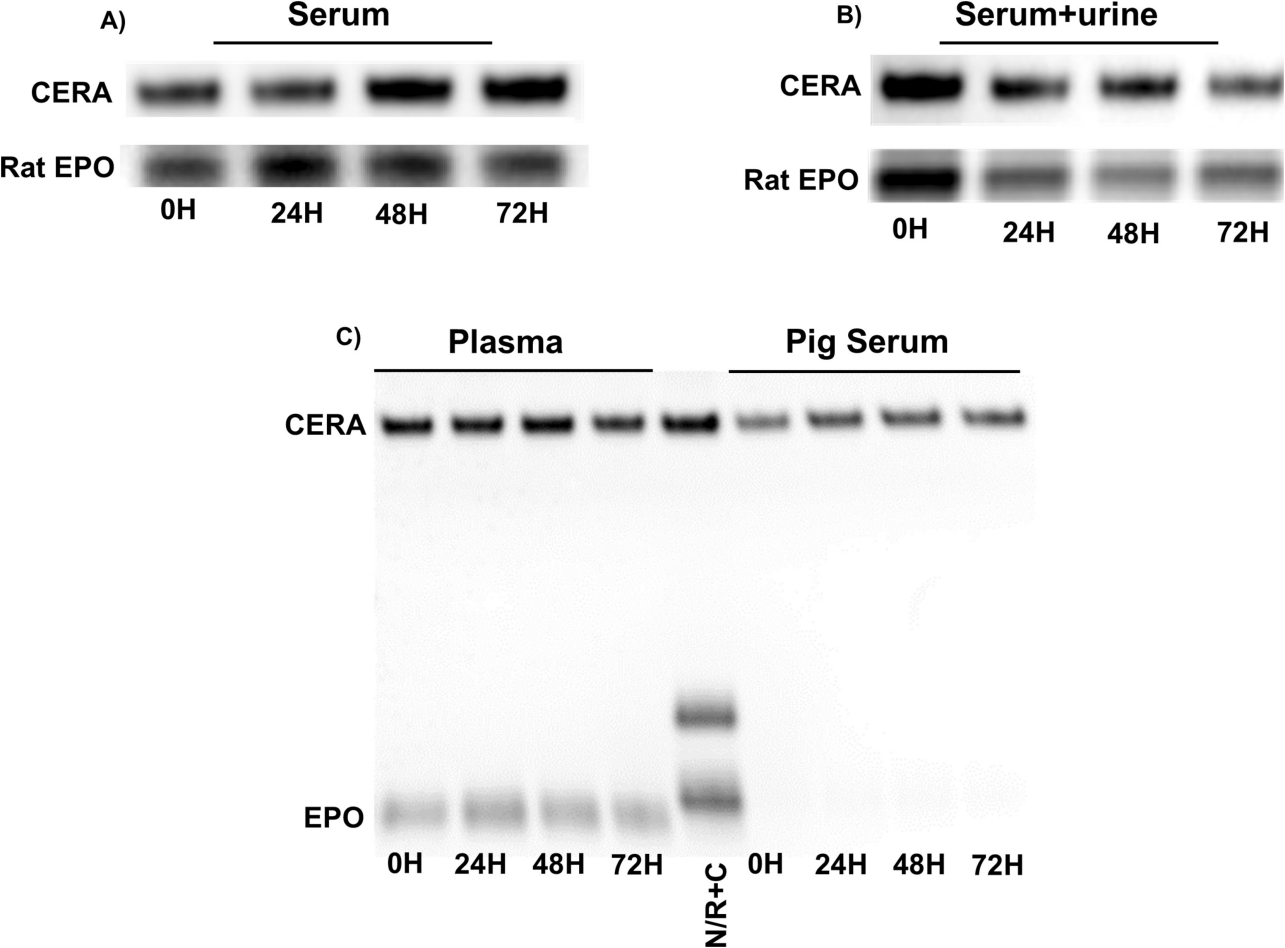
CERA stability in serum and plasma under thermal stress. (A) Serum samples spiked with CERA were incubated at 37°C and analyzed at 0, 24, 48, and 72 h. (B) CERA‐containing serum was added to an unstable urine sample to evaluate potential stabilization. (C) CERA‐spiked plasma and pig serum were similarly incubated and analyzed over time. Rat EPO was used as an internal standard.

Conversely, the spiked serum sample demonstrated exceptional stability of CERA under storage conditions at 37°C (Figure 4A). The stability of ERAs in serum has already been demonstrated for other erythropoiesis‐stimulating agents [21]. To evaluate whether serum‐specific components could mitigate the degradation of CERA in urine under elevated temperatures, CERA‐spiked human serum was added to the female urine sample that had previously shown the most pronounced degradation. Following incubation at 37°C, the CERA signal remained stable, suggesting that constituents present in serum may exert a protective effect against CERA degradation, potentially by inhibiting enzymatic activity or stabilizing the molecule's structure (Figure 4B). These findings support the hypothesis that specific serum proteins, such as albumin or S100 proteins, which have previously been reported to stabilize CERA [22, 23], may interact with the molecule to preserve its structural integrity and protect it from degradation under compromised conditions.

To further investigate whether this stabilizing effect was specific to human serum, additional analyses were conducted using human plasma and pig serum (Figure 4C). As expected, human plasma maintained CERA stability at 37°C, consistent with the preservation observed in serum. Interestingly, pig serum also preserved CERA stability under the same conditions, despite the absence of detectable endogenous EPO in this matrix (Figure 4C). This finding is practically relevant, as pig serum is substantially more cost‐efficient than human serum and may serve as a suitable neutral matrix for preparing quality control samples. Taken together, these findings highlight the critical influence of biological matrix composition on CERA stability. They suggest that specific protein‐based interactions, which likely involve abundant serum proteins, may play a protective role against degradation. In contrast, the absence or lower concentration of such stabilizing components in urine, particularly in samples with higher microbial content, may contribute to the reduced stability. These results stress the importance of selecting the right matrix and composition, both in developing analytical methods and in the interpretation of CERA results.

**FIGURE 5:**
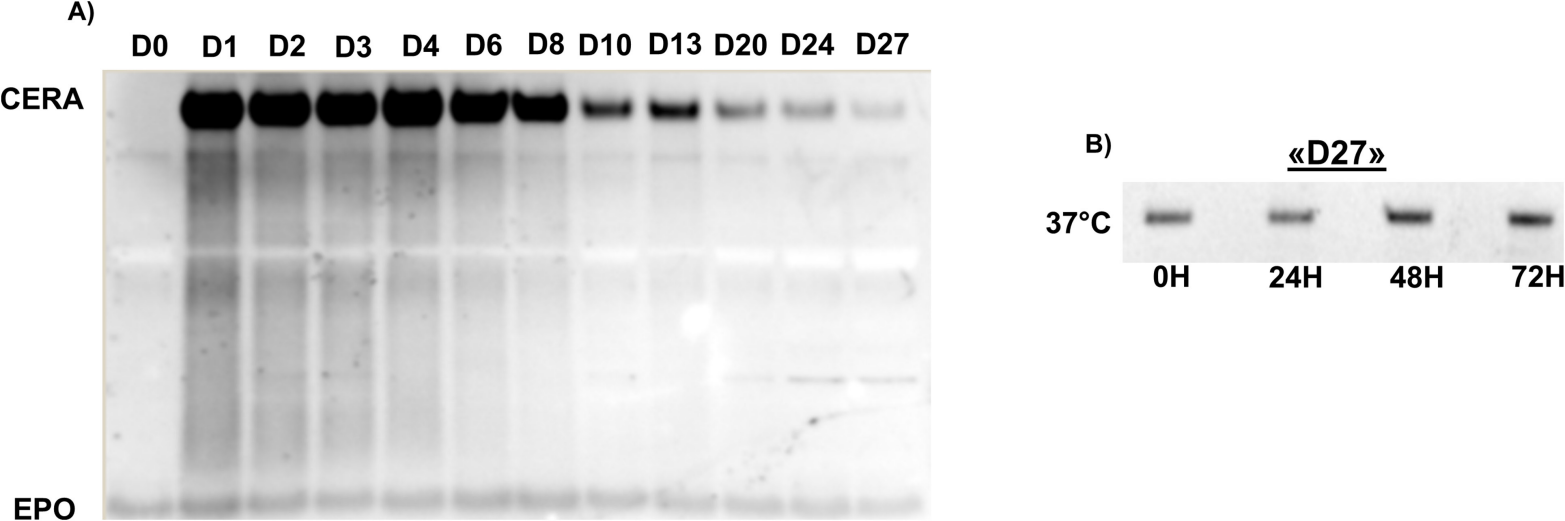
CERA remains stable in dried blood spots (DBS) under stress conditions*.* (A) Modeled Tasso‐M20 samples from a volunteer administered with 200 μg of CERA were analyzed up to Day 27 post‐injection. D0 corresponds to pre‐injection time points. (B) DBS collected on Day 27 were incubated at 37°C and analyzed at 0, 24, 48, and 72 h to assess thermal stability.

To further explore the potential of alternative matrices for CERA detection, we evaluated both the molecular stability and detection window of CERA in DBS created using Tasso‐M20 samples. This included an assessment of long‐term detectability of CERA on modeled Tasso‐M20 samples from one subject selected from a previous administration study, in which six subjects received a single 200 μg dose of CERA [11]. Using SAR‐PAGE analysis, CERA was detectable in the matrix for up to 27 days post‐administration (Figure 5A), demonstrating an extended detection window despite the small sample volume. Additionally, Tasso‐M20 samples prepared from serum collected on Day 27 (D27) were incubated at 37°C to evaluate CERA stability under conditions previously shown to promote degradation in urine. Similar to serum, no degradation was visible in the D27 Tasso‐M20 sample, confirming the stability of CERA in the DBS matrix. These results align with previous findings on the stability of ERAs in DBS, further supporting the suitability of Tasso‐M20 devices for anti‐doping purposes, especially in scenarios involving extended transport times [24]. Together with its demonstrated ability to preserve CERA under compromised conditions, the minimally invasive Tasso‐M20 and Tasso+ devices hold promise for longitudinal monitoring of hematological and steroid biomarkers within the ABP [25, 26]. By complementing traditional blood matrices, capillary blood collection could enhance both direct ERA detection and indirect biomarker‐based profiling.

## 4 Conclusion

Within the framework of the ABP, the prolonged detectability of CERA through direct analytical methods provides a valuable complement to the indirect markers, which reflect distinct physiological responses. While the erythropoietic effect is first evidenced by an increase in reticulocytes typically 5–7 days after CERA administration, the rise in hemoglobin levels follows a slower kinetic, generally occurring after 10 days. Therefore, timely target testing and careful selection of both the biological matrix and type of analysis are crucial to maximize the success of ERA detection, particularly for substances like CERA with unique pharmacokinetic and elimination properties. The case study presented herein highlights important differences in CERA detectability and stability across biological matrices. In this specific example, CERA was consistently detected in serum samples, while matched urine specimens yielded negative results, likely as a consequence of its PEGylated high molecular weight, which limits renal excretion. These findings reinforce the use of blood, particularly serum, as the preferred matrix for CERA detection.

Additionally, pronounced instability of CERA was observed in human urine, potentially due to matrix‐specific degradation mechanisms, whereas serum and plasma provided greater stability, even at the challenging temperature of 37°C. Despite low volume, dried blood samples (Tasso‐M20) also demonstrated excellent analyte preservation and an extended detection window (up to 27 days), supporting their applicability to out‐of‐competition and decentralized testing in remote locations. Taken together, these results reinforce the importance of selecting appropriate matrices for ERA detection and support the continued integration of micro‐sampling devices in anti‐doping programs.

## Conflicts of Interest

The authors declare no conflicts of interest.

## Data Availability

The data that support the findings of this study are available from the corresponding author upon reasonable request.

## References

[1] O. Salamin , T. Kuuranne , M. Saugy , and N. Leuenberger , “Erythropoietin as a Performance‐Enhancing Drug: Its Mechanistic Basis, Detection, and Potential Adverse Effects,” Molecular and Cellular Endocrinology 464 (2018): 75–87. 28119134 10.1016/j.mce.2017.01.033

[2] C. Reichel , F. Abzieher , and T. Geisendorfer , “SARCOSYL‐PAGE: A New Method for the Detection of MIRCERA‐ and EPO‐Doping in Blood,” Drug Testing and Analysis 1, no. 11–12 (2009): 494–504. 20355164 10.1002/dta.97

[3] A. Marchand , C. Buisson , L. Martin , J.‐A. Martin , A. Molina , and D. Ressiot , “Report on an Anti‐Doping Operation in Guadeloupe: High Number of Positive Cases and Inferences About Doping Habits,” Drug Testing and Analysis 9, no. 11–12 (2017): 1753–1761. 28296276 10.1002/dta.2185

[4] Y. O. Schumacher , M. Saugy , T. Pottgiesser , and N. Robinson , “Detection of EPO Doping and Blood Doping: The Haematological Module of the Athlete Biological Passport,” Drug Testing and Analysis 4, no. 11 (2012): 846–853, 10.1002/dta.406. 22374784

[5] C. Schobinger , C. Emery , C. Schweizer‐Gründisch , and T. Kuuranne , “Support of a Laboratory‐Hosted Athlete Biological Passport Management Unit (APMU) to the Anti‐Doping Organisations,” Rechtsmedizin 31 (2021): 526–532, 10.1007/s00194-021-00456-x.

[6] R. Faiss , J. Saugy , and M. Saugy , “Fighting Doping in Elite Sports: Blood for All Tests!,” Frontiers in Sports and Active Living 1 (2019): 30, 10.3389/fspor.2019.00030. 33344954 PMC7739585

[7] C. E. Heiland , L. Martin , X. Zhou , L. Zhang , M. Ericsson , and A. Marchand , “Dried Blood Spots for Erythropoietin Analysis: Detection of Micro‐Doses, *EPO* c.577del Variant and Comparison With In‐Competition Matching Urine Samples,” Drug Testing and Analysis 16, no. 6 (2024): 650–654. 37942506 10.1002/dta.3596

[8] G. D. Miller , J. M. Goodrum , A. K. Flores , A. K. Crouch , and D. Eichner , “Detecting EPO in Microvolumetric Capillary Serum Shipped at Ambient Temperature for Antidoping Testing,” Drug Testing and Analysis 17 (2024): 1232–1236, 10.1002/dta.3831. 39552447

[9] World Anti Doping Agency (WADA) , “Technical Document for the Harmonization of Analysis and Reporting of Erythropoietin (EPO)‐Receptor Agonists (ERAs) and Transforming Growth Factor‐Beta (TGF‐b) Signalling Inhibitors by Polyacrylamide Gel Electrophoretic (PAGE) Analytical Methods,” (TD2024EPO). World Anti Doping Agency 2024, accessed August 5, 2025, https://www.wada‐ama.org/en/resources/lab‐documents/td2024epo.

[10] X. Zhou , L. Zhang , S. He , L. Shen , and C. He , “Comparison and Optimization of SAR‐PAGE Tests for Erythropoietins in Doping Analysis,” Drug Testing and Analysis 12, no. 1 (2020): 109–118. 31668004 10.1002/dta.2703

[11] S. Lamon , S. Giraud , L. Egli , et al., “A High‐Throughput Test to Detect C.E.R.A. Doping in Blood,” Journal of Pharmaceutical and Biomedical Analysis 50, no. 5 (2009): 954–958. 19625154 10.1016/j.jpba.2009.06.038

[12] A. Rocca , L. Martin , T. Kuuranne , M. Ericsson , A. Marchand , and N. Leuenberger , “A Fast Screening Method for the Detection of CERA in Dried Blood Spots,” Drug Testing and Analysis 14, no. 5 (2022): 820–825. 34380180 10.1002/dta.3142 PMC9540874

[13] G. Reverter‐Branchat , R. Ventura , M. Ezzel Din , J. Mateus , C. Pedro , and J. Segura , “Detection of Erythropoiesis‐Stimulating Agents in a Single Dried Blood Spot,” Drug Testing and Analysis 10, no. 10 (2018): 1496–1507. 29877055 10.1002/dta.2418

[14] X. Zhou , S. He , L. Zhang , L. Shen , and C. He , “Research on Spiking Rat EPO as Internal Standard in Doping Control Samples for Detection of EPO Using SAR‐PAGE Analysis With Biotinylated Primary Antibody,” Drug Testing and Analysis 12, no. 8 (2020): 1054–1064. 32449841 10.1002/dta.2863

[15] Cyclingnews , “Vuelta a Costa Rica Winner Among 12 Riders to Test Positive in 2017 edition|Cyclingnews.com,” Cyclingnews 2018, accessed August 5, 2025, https://www.cyclingnews.com/news/vuelta‐a‐costa‐rica‐winner‐among‐12‐riders‐to‐test‐positive‐in‐2017‐edition/.

[16] Y. Dehnes and P. Hemmersbach , “Effect of Single Doses of Methoxypolyethylene Glycol‐Epoetin Beta (CERA, Mircera) and Epoetin Delta (Dynepo) on Isoelectric Erythropoietin Profiles and Haematological Parameters,” Drug Testing and Analysis 3, no. 5 (2011): 291–299. 21387570 10.1002/dta.270

[17] F. Loria , A. P. Stutz , A. Rocca , et al., “Monitoring of Hemoglobin and Erythropoiesis‐Related mRNA With Dried Blood Spots in Athletes and Patients,” Bioanalysis 14, no. 5 (2022): 241–251. 35172618 10.4155/bio-2021-0252

[18] J. Bejder , N. J. Aachmann‐Andersen , T. C. Bonne , N. V. Olsen , and N. B. Nordsborg , “Detection of Erythropoietin Misuse by the Athlete Biological Passport Combined With Reticulocyte Percentage,” Drug Testing and Analysis 8, no. 10 (2016): 1049–1055. 27696774 10.1002/dta.1932

[19] F. Lasne , L. Martin , J. A. Martin , and J. de Ceaurriz , “Detection of Continuous Erythropoietin Receptor Activator in Blood and Urine in Anti‐Doping Control,” Haematologica 94, no. 6 (2009): 888–890. 19483162 10.3324/haematol.2009.006809 PMC2688586

[20] N. Robinson , P.‐E. Sottas , and M. Saugy , “Fluorescence Flow Cytometer to Determine Urine Particle Reference Intervals in Doping Control Samples,” Forensic Science International 213, no. 1–3 (2011): 95–100. 21889276 10.1016/j.forsciint.2011.07.054

[21] G. D. Miller , J. M. Goodrum , A. K. Crouch , and D. Eichner , “Assessing EPO Stability in Urine and Comparing Recombinant EPO Detectability in Matched Urine, Venous Serum, and Capillary Serum Following a Controlled Epoetin Alfa Administration,” Drug Testing and Analysis 17 (2025): 358–364, 10.1002/dta.3736. 38785206

[22] J. He , M. Feng , X. Zhou , et al., “Stabilization and Encapsulation of Recombinant Human Erythropoietin Into PLGA Microspheres Using Human Serum Albumin as a Stabilizer,” International Journal of Pharmaceutics 416, no. 1 (2011): 69–76. 21699969 10.1016/j.ijpharm.2011.06.008

[23] A. S. Kazakov , E. I. Deryusheva , A. S. Sokolov , et al., “Erythropoietin Interacts With Specific S100 Proteins,” Biomolecules 12, no. 1 (2022): 120. 35053268 10.3390/biom12010120 PMC8773746

[24] L. Requena‐Tutusaus , I. Anselmo , É. Alechaga , R. Bergés , and R. Ventura , “Achieving Routine Application of Dried Blood Spots for Erythropoietin Receptor Agonist Analysis in Doping Control: Low‐Volume Single‐Spot Detection at Minimum Required Performance Level,” Bioanalysis 15 (2023): 1235–1246, 10.4155/bio-2023-0118. 37676639

[25] J. M. Goodrum , L. A. Lewis , M. N. Fedoruk , D. Eichner , and G. D. Miller , “Feasibility of Microvolumetric Capillary Whole Blood Collections for Usage in Athlete Biological Passport Analysis,” Drug Testing and Analysis 14, no. 7 (2022): 1291–1299. 35302295 10.1002/dta.3254

[26] J. M. Goodrum , K. Peek , C. Moore , D. Eichner , and G. D. Miller , “Is Blood Blood? Comparing Quantitation of Endogenous Steroids and Luteinizing Hormone in Concurrently Collected Venous Serum and Tasso+ SST Capillary Serum Samples,” Drug Testing and Analysis 17, no. 3 (2025): 365–371. 38794805 10.1002/dta.3738

